# Molecular typing and epidemiologic profiles of human metapneumovirus infection among children with severe acute respiratory infection in Huzhou, China

**DOI:** 10.1007/s11033-021-06776-1

**Published:** 2021-10-19

**Authors:** Lei Ji, Liping Chen, Deshun Xu, Xiaofang Wu

**Affiliations:** Huzhou Center for Disease Control and Prevention, Huzhou, Zhejiang Province China

**Keywords:** Human metapneumovirus, Respiratory tract infection, Epidemiology

## Abstract

**Background:**

Human metapneumovirus (hMPV) is one of the important pathogens in infant respiratory tract infection. However, the molecular epidemiology of hMPV among children < 14 years of age hospitalized with severe acute respiratory infection (SARI) is unclear. We investigated the hMPV infection status and genotypes of children hospitalized with SARI from January 2016 to December 2020 in Huzhou, China.

**Methods:**

A nasopharyngeal flocked swab, nasal wash, or nasopharyngeal swab/or opharyngeal swab combination sample was collected from children with SARI in Huzhou from January 2016 to December 2020. Quantitative reverse transcription-polymerase chain reaction was performed to detect hMPV RNA. The hMPV F gene was amplified and sequenced, followed by analysis using MEGA software (ver. 7.0). Epidemiological data were analyzed using Microsoft Excel 2010 and SPSS (ver. 22.0) software.

**Results:**

A total of 1133 children with SARI were recruited from 2016 to 2020. Among them, 56 (4.94%) were positive for hMPV-RNA. Children < 5 years of age accounted for 85.71% of the positive cases. The hMPV incidence was high in spring and winter, especially in December and January to March. Phylogenetic analysis of the F-gene sequences of 28 hMPV strains showed that the A1, B1, and B2 genotypes were prevalent in Huzhou, and the dominant hMPV genotype varied according to surveillance year.

**Conclusions:**

HMPV is an important respiratory pathogen in children in Huzhou, with a high incidence in winter and spring in children < 5 years of age. In this study, genotypes A1, B1, and B2 were the most prevalent.

## Background

Human metapneumovirus (hMPV), first identified in 2001, is a major viral respiratory pathogen worldwide [1–3]. hMPV is an important pathogen in infant respiratory tract infection—about half of all children are infected by hMPV before 2 years of age, and most are infected before 5 years of age [1]. In southern China, 2.0% of hospitalized children < 14 years of age were positive for hMPV, and the prevalence was higher in those aged ≤ 5 years (2.2%) than those aged 5–14 years(0.7%) [4]. Unfortunately, individuals infected with hMPV typically do not develop lifelong immunity, and reinfection occurs frequently [1, 5]. hMPV can cause fatal complications in young children, the elderly, and the immunocompromised [6]. Global epidemiological studies have shown that most children have been infected with hMPV by the age of 5 years [1]. hMPV infection cannot be distinguished from other respiratory viral infections by its symptoms alone. hMPV can cause upper or lower respiratory tract infection, mainly manifesting as cough, expectoration, wheezing, shortness of breath, runny nose, bronchitis, asthmatic bronchitis, bronchiolitis, and pneumonia [7]. hMPV is a non-segmented, negative-stranded RNA virus of the family Pneumoviridae. The viral RNA is approximately 13 kb in length, and contains eight genes (N, P, M, F, M2, SH, G, and L) coding for nine proteins [7]. Evolutionary analysis of multiple genes showed that hMPV comprises genotypes A and B, which both have two subtypes (A1 and A2; and B1 and B2, respectively) [8]. The fusion protein encoded by the F gene is the main antigen of hMPV, and is typically used for typing [9]. Different hMPV subtypes can be prevalent simultaneously, and the dominant types can change over time [10]. The major prevalent subtypes are A2, B1, and B2 [11–13]. The correlation of hMPV subtypes with disease severity is inconsistent [14–16]. hMPV infection is increasingly being reported in Asian countries [11, 17–23], with novel variants of hMPV also emerging [22, 23], highlighting the risk of hMPV epidemics in these regions. However, there is no effective vaccine or antiviral for hMPV [24]. Therefore, it is important to understand the prevalence and pathogenesis of hMPV for prevention and treatment. Analysis of the genomic structure of hMPV genotypes would provide insight into its genetic background and evolutionary status, and facilitate epidemiological investigation, prevention, and treatment of hMPV.

The genetic evolution and transmission of hMPV are critical for epidemic control. We conducted a 5-year study on hMPV in Huzhou, the most populous city in China, from January 2016 to December 2020.

## Materials and methods

### Patients and clinical samples

Patients suspected of having acute respiratory tract infections were enrolled according to these criteria [25]: a history of fever(> 38 °C) accompanied by sore throat, cough, expectoration, or dyspnea/tachypnea, for no longer than 10 days. Samples were obtained from the local severe acute respiratory infection (SARI) surveillance sentinel hospital, the First People’s Hospital of Huzhou. A total of 1133 patients were enrolled from January 2016 to December 2020. A nasopharyngeal flocked swab, nasal wash or combination nasopharyngeal swab/or opharyngeal swab sample was collected from each patient, placed in 3 mL of viral transport medium, and stored at − 80 °C prior to laboratory screening.

### RNA extraction and quantitative reverse transcription-polymerase chain reaction

Viral RNA was extracted from 200 μL of clinical specimens using a QIAamp Viral RNA Mini Kit (Qiagen, Hilden, Germany) according to the manufacturer’s instructions. The RNA extracts were subjected to reverse transcription polymerase chain reaction (RT-PCR) or stored at − 70 °C. Real-time quantitative RT-PCR (qRT-PCR) was performed using a Nucleic Acid Detection Kit for hMPV (Zhuocheng, Beijing, China) with the ABI Q7 (Applied Biosystems, Foster City, CA, USA). The reaction was conducted according to the manufacturer’s instructions in a total volume of 20 μL.

### PCR amplification of F-protein genes

For hMPV-positive samples, the viral RNA was reverse-transcribed into cDNA using the One-Step RNA PCR Kit (TaKaRa Biotechnology, Dalian, China). The F gene was amplified by traditional PCR using primers reported previously [26]. The primers for F-gene sequencing were outer hMPV-F-F1-5′-CAATGCAGGTATAACACCAGCAATATC-3′ and hMPV-F-R1 5′-GCAACAATTGAACTGATCTTCAGGAAAC-3′, and inner hMPV-F-F2-5′-ACATGCCAACATCTGCAGGACAAATAAAAC-3′ and hMPV-F-R2 5′-ACATGCTGTTCACCTTCAACTTTGC-3′. The primers were designed to amplify the 596 bp region between nucleotides 3749 and 4344 (reference strain: AF371337). The amplification conditions were 95 °C for 5 min, followed by 35 cycles of 95 °C for 30 s,57 °C for 30 s, and 72 °C for 1.5 min, and a final step at 72 °C for 5 min. Next, 5 μL of the PCR products were resolved by 2% agarose gel electrophoresis. The residual PCR products were purified using a QIAQ uick PCR Purification Kit (Qiagen), and the purified products were sequenced directly at both ends using amplification primers by TaKaRa Biotechnology.

### Phylogenetic analysis

hMPV A1, B1, and B2 reference sequences (LC337720, GU048745, MT118718, LC337745, GU048746, JQ745069, KF192752, GU048745, and GU048741) were downloaded from GenBank. Phylogenetic analysis was performed by the neighbor-joining algorithm and Kimura two-parameter model, supported by bootstrapping with 1000 replicates in MEGA software (ver. 7.0).

### Statistical analysis

Microsoft Excel 2010 (Microsoft Corp, Redmond, WA, USA and SPSS (ver. 22.0; SPSS Inc., Chicago, IL, USA) software were used for statistical analysis. The chi-squared test was used for comparisons between groups. P values < 0.05 indicate significant differences.

### Nucleotide sequence accession numbers

The GenBank accession numbers for the sequences obtained in this study are MZ215789–MZ215816.

## Results

### Epidemic characteristics of hMPV

A total of 1133 nasopharyngeal swab samples were collected from hospitalized children with SARI from January 2016 to December 2020; 56 samples were positive for hMPV, for a detection rate of 4.94% (56/1,133). There was no significant difference in the positivity rate between males and females (P = 0.395). The hMPV positivity rate was 6.03% 4.43%, 4.61%, 4.76%, and 4.39% in the 0-, 1-,3-,5-, and 7–14-year-old groups, respectively. There were no significant differences in hMPV detection rates among the age groups (P = 0.890) (Table 1).

**Table 1:** hMPV-positive in Pediatric Patients of different ages and gender with SARI

Variable	Tested SARI cases N (percentage)	hMPV-positive cases N(percentage)	hMPV-negative cases N (percentage)	Positive rate (%)	χ^2^	P
Gender					0.724	0.395
Male	605 (53.40)	33 (58.93)	572(53.11)	5.45		
Female	528 (46.60)	23 (41.07)	505(46.29)	4.36		
Age (years)					1.126	0.890
0 ~	315 (27.80)	19 (33.93)	296 (27.48)	6.03		
1 ~	361 (31.86)	16 (28.57)	345 (32.03)	4.43		
3 ~	282 (24.89)	13 (23.21)	269 (24.98)	4.61		
5 ~	84 (7.41)	4 (7.14)	80 (7.42)	4.76		
7 ~ 14	91 (8.03)	4 (7.14)	87 (8.08)	4.39		
Total	1133	56	1077	4.94		

**Fig. 1:**
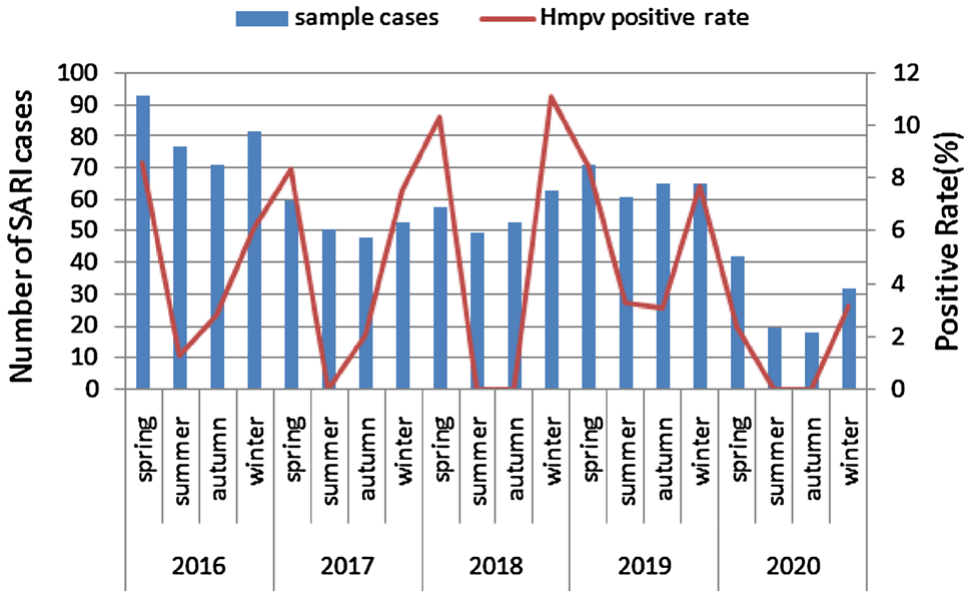
Seasonal distribution of hMPV infection from 2016 to 2020

In 2016–2019, the hMPV detection rate was 4.72–5.80%, similar to central and south China. The rate of hMPV was low in 2020, with a positivity rate of 1.79%. hMPV circulated predominantly in spring and winter (Fig. 1). In contrast, the hMPV infection rate was lower in summer and autumn(1.16% and 1.96%, respectively).

### HMPV genotyping and phylogenetic analysis

**Fig. 2:**
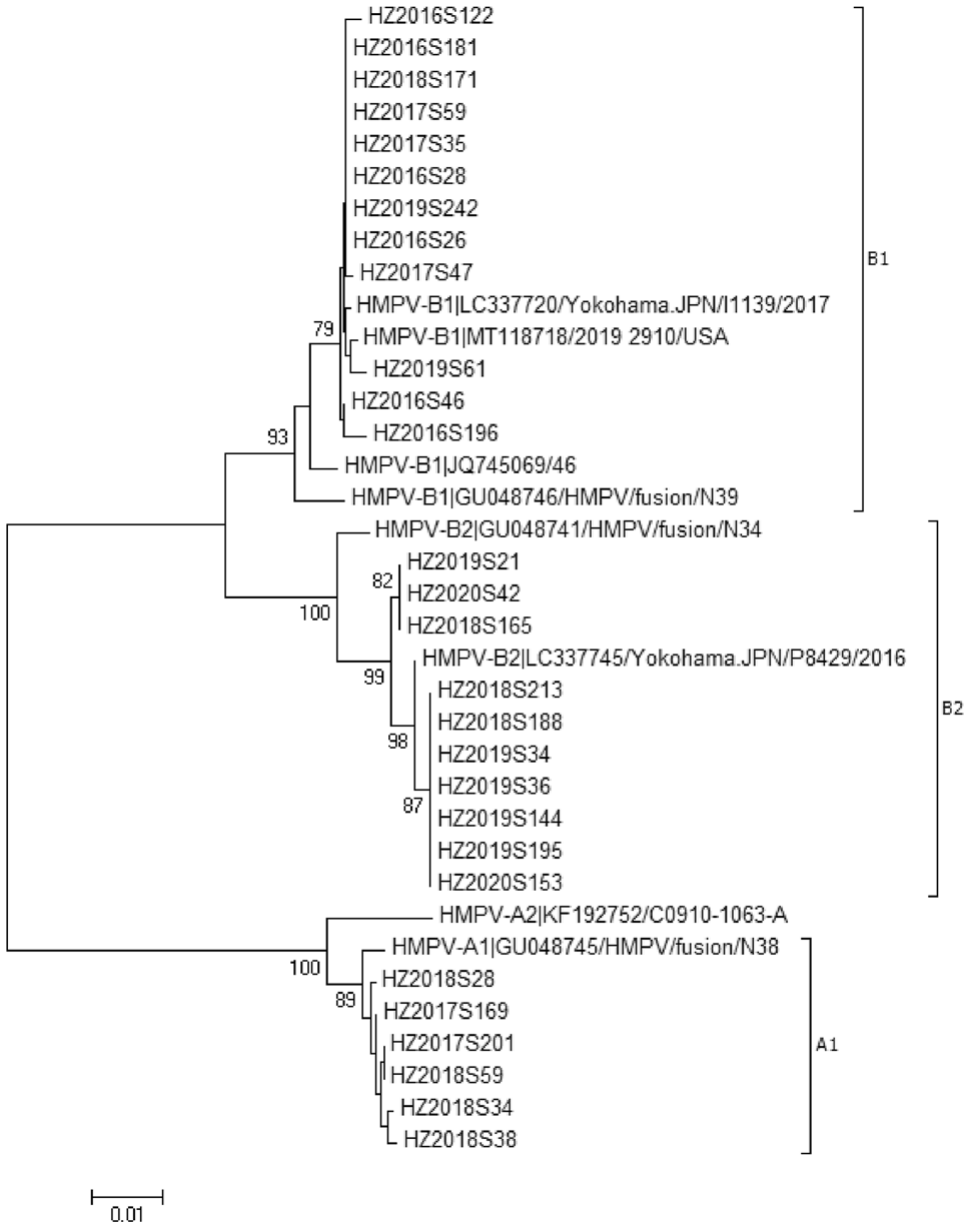
Genotyping and phylogenetic analysis of hMPV strains. Trees were generated using the neighbor-joining method, validated by 1000 bootstrap replicates. Bootstrap values ≥ 70% are shown on branches. The hMPV sequences identified in this study are indicated by closed circles

F-gene amplification and sequencing were performed on 56hMPV-positive samples, and the F-gene sequences of 28 strains were obtained. Phylogenetic analysis of reference strains and those obtained in this study (MZ215789–MZ215816) was performed using MEGA software (ver. 7.0) with 1000 bootstrap replicates. Six strains were A1 genotype, 12 were B1, and 10 strains were B2.The predominant genotypes of hMPV varied according to surveillance year. One type (hMPV-B1) was detected in 2016, two in 2017 [hMPV-B1 (n = 3) and HAdV-A1 (n = 2)], three in 2018 [hMPV-B1 (n = 1), hMPV-B2 (n = 3), and hMPV-A1 (n = 4)], two in 2019 [hMPV-B1 (n = 2) and hMPV-B2 (n = 5)], and one (hMPV-B2) in 2020 (Fig. 2).

## Discussion

hMPV is an important respiratory pathogen. The diseases caused by hMPV are not markedly different from other viral infections, ranging from mild upper respiratory tract infection to severe bronchopneumonia. The clinical manifestations are cough, runny nose, fever, and wheezing. Hypoxemia occurs in about one-third of patients. Chest X-Ray shows local infiltrating shadows on the pulmonary lobes or infiltration around the hilar lung and peritracheal cuff sign. It is estimated that 4–16% of acute respiratory tract infections are caused by hMPV [27, 28], which was first identified in 2001. In 2003, Runan et al. [12] reported a case of hMPV infection in China, and other cases followed [13, 17].

hMPV infection is seasonal, with a peak in winter and spring. In the northern hemisphere, hMPV disease occurrence peaks typically in winter and spring (January to May) [29–31], while in the southern hemisphere peak prevalence is in the period August–September [32]. In this study, the hMPV detection rate differed among months. The peak months were November and January to March; thus, the epidemic season was winter and spring, consistent with Yu [18]. In addition, the hMPV detection rate was 4.72–5.80% in 2016–2019, but low in 2020 (1.79%), possibly because of the emergence of severe acute respiratory syndrome coronavirus-2 in February 2020 in Huzhou, increased awareness of crowd protection, restrictions on crowd gathering, and suspension of kindergartens and schools.

Worldwide, the hMPV prevalence in hospital inpatient and community studies, in children and the elderly, varies from 1.7 to 17%, with a higher prevalence in outpatients compared to inpatients, and in children < 5 years of age compared to older age groups [29, 33, 34], as in other parts of China [4, 10]. In this study, 1133 children with severe acute respiratory tract infection in Huzhou from 2016 to 2020 were tested for hMPV RNA, and 56 (4.94%) were positive (negative for other pathogens), indicating that hMPV is an important respiratory pathogen among children in Huzhou. The proportion of cases < 5 years of age was 84.55% (958/1133), and that of positive cases was 85.71% (48/56). There was no significant sex difference in infection rate, as also reported by Meijia [19].

hMPV genotypes A and B can be prevalent together in the same season, although genotype A is the most prevalent. The prevalences of hMPV genotypes in the same region may change annually. Shiwen reported [10] that hMPV genotypes A2, B1, and B2 were prevalent in Jiangxi, among which A2 predominated. hMPV genotypes A1, B1, and B2 were prevalent in the Huzhou area. The B1 genotype was the most prevalent, and was detected every year (except 2020), followed by B2 and A1, indicating that B1 is the main epidemic strain in Huzhou. However, the genotypes of the endemic strains differ from year to year, and several endemic types exist simultaneously every year. No hMPV genotype predominated during the hMPV epidemic season.

This study was limited by the single-site setting, small sample size, relationship between disease severity and genotype, and partial genotyping of hMPV. Genotyping was successful for only 50% (28/56) of hMPV infection cases. We plan to increase the detection range and quantity of samples, evaluate the harm caused by hMPV-related diseases, and provide a scientific basis for the prevention and control of hMPV infection.

## Conclusions

HMPV is an important pathogen of SARI in hospitalized children in Huzhou. It usually occurs in winter and spring in children < 5 years of age. hMPV genotypes A1, B1, and B2 were prevalent, but none predominated during the hMPV epidemic season, hampering the diagnosis and treatment of SARI. Our results will improve the prevention and control of hMPV in Huzhou.

## Data Availability

The readers interested in using the data may contact the corresponding author.

## Abbreviations

SARI: Severe acute respiratory infections
hMPV: Human metapneumovirus
qPCR: Real-time RT-PCR
